# Spatiotemporal dynamics of brain function during the natural course in a dental pulp injury model

**DOI:** 10.1007/s00259-022-05764-2

**Published:** 2022-03-19

**Authors:** Feiyan Yu, Miao Li, Qianqian Wang, Jing Wang, Shuang Wu, Rui Zhou, Han Jiang, Xiaoyi Li, Yu Zhou, Xi Yang, Xiao He, Yan Cheng, Xiuyun Ren, Hong Zhang, Mei Tian

**Affiliations:** 1 Department of Periodontology, Shanxi Province Key Laboratory of Oral Diseases Prevention and New Materials, Shanxi Medical University School and Hospital of Stomatology, No. 63, New South Road, Yingze District, Taiyuan, 030001 Shanxi China; 2 Department of Nuclear Medicine and PET Center, The Second Affiliated Hospital of Zhejiang University School of Medicine, 88 Jiefang Road, Hangzhou, 310009 Zhejiang China; 3 PET-CT Center, Fujian Medical University Union Hospital, Fuzhou, 350001 Fujian China; 4 Institute of Nuclear Medicine and Molecular Imaging of Zhejiang University, Hangzhou, 310009 Zhejiang China; 5 Key Laboratory of Medical Molecular Imaging of Zhejiang Province, Hangzhou, 310009 Zhejiang China; 6 First Hospital of Shanxi Medical University, Taiyuan, 030001 Shanxi China; 7 Human Phenome Institute, Fudan University, Shanghai, 201203 China

**Keywords:** Toothache, Positron emission tomography (PET), Dental pulp injury (DPI), Caudal anterior cingulate cortex (cACC)

## Abstract

**Purpose:**

Toothache, a common disorder afflicting most people, shows distinct features at different clinical stages. This study aimed to depict metabolic changes in brain and investigate the potential mechanism involved in the aberrant affective behaviors during the natural process of toothache.

**Methods:**

We investigated the spatiotemporal patterns of brain function during the natural course of toothache in a rat model of dental pulp injury (DPI) by using positron emission tomography (PET).

**Results:**

Glucose metabolism peaked on the 3rd day and gradually decreased in several brain regions after DPI, which was in line with the behavioral and histological results. PET imaging showed that visual pathway was involved in the regulation of toothache. Meanwhile, the process of emotional regulation underlying toothache was mediated by N-methyl-D-aspartic receptor subunit 2B (NR2B) in the caudal anterior cingulate cortex (cACC).

**Conclusion:**

Our results revealed the spatiotemporal neurofunctional patterns during toothache process and preliminarily elucidated the role of NR2B in cACC in the regulation of toothache-related affective behaviors.

**Supplementary Information:**

The online version contains supplementary material available at 10.1007/s00259-022-05764-2.

## Introduction

Toothache is considered to be the highest in the degree of pain and often leads to some emotional symptoms such as anxiety- and depression-related behaviors [1]. Many studies have confirmed that the neural conduction pathway of the toothache is the trigeminal nerve-thalamo-cortical circuitry, which included the brainstem trigeminal complex, thalamus, and cerebral cortex [2, 3]. The signal of nociceptive stimulus in the dental pulp is transmitted from the periphery to the central nervous system through the trigeminal ganglion, and then converges to the trigeminal spinal nucleus, which relays nociceptive information to the thalamus, and further transduces nociceptive information to the cerebral cortex [2–4]. However, the relationship between toothache and emotional reaction is not clear. The dynamic spatio-temporal changes of brain function involved in the natural process of toothache also have not been clearly studied. Here, for the first time to our knowledge, we non-invasively investigated the dynamic metabolic changes of brain activities longitudinally using PET molecular imaging approach [5] in a rat model of dental pulp injury (DPI), and further explored the potential mechanisms underlying the aberrant affective behaviors during the toothache natural course.

## Materials and methods

### Animals

Adult male Sprague–Dawley (SD) rats (240–260 g) (*n* = 60) were randomly assigned to the DPI group and the sham group for natural process investigation (1st, 2nd, 3rd, 7th, 14th day, respectively, total of 10 groups, *n* = 6 in each group). For intervention study, the rats (*n* = 48) were randomly assigned to four groups (including the sham, DPI, DPI + NS and DPI + APV treated, *n* = 12 in each group). We performed DPI on part of the rats by mechanically exposing the dental pulp as previously described [6], which produces pulpal inflammation (pulpitis) followed by necrosis of the pulpal tissues. A double-guide cannula was embedded into the cACC [7], then rats were allowed to recover for 7–10 days before intervention to evaluate the wound healing and weight recovery. All the procedures are provided in the Supplementary Material.

### Behavioral assessment

**Fig. 1:**
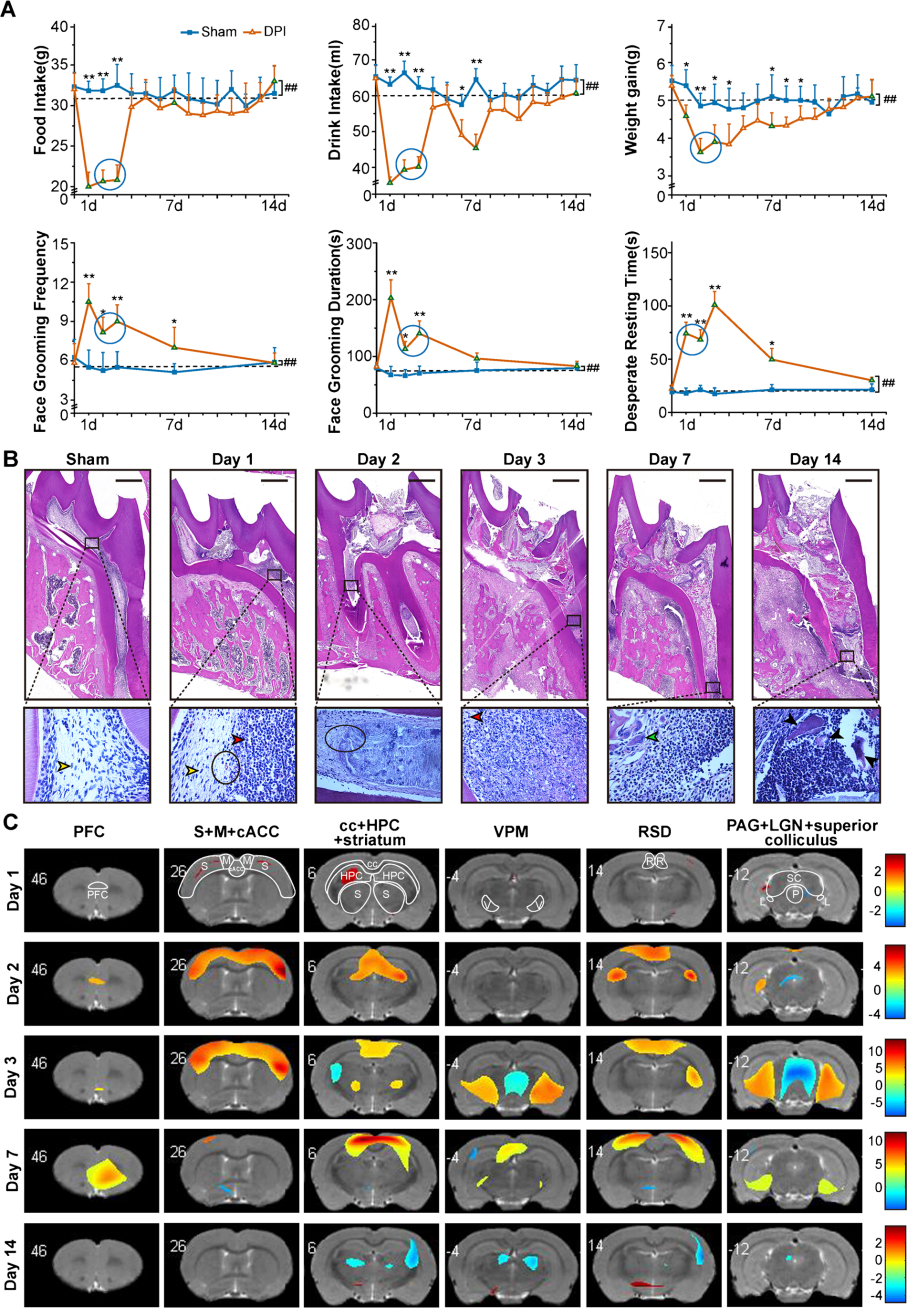
Establishment of DPI verified by behavioral assessment, Histology and PET molecular imaging. **A** Behavioral changes of food intake, drink intake, average weight gain, face grooming frequency, face grooming time, and freezing time in DPI. **B** HE staining images showed the development changes of pulpitis during the natural course of DPI (bar = 250 μm). The yellow arrows represent normal pulp cells, the red arrows indicate neutrophils, the green arrows indicate osteoclasts, the black circles represent the border of normal pulp and inflammatory tissue, and the black arrows indicate necrotic tooth tissue (400 ×). **C** Brain regions that showed significant glucose metabolism changes on days 1, 2, 3, 7, and 14 after DPI operation in rats (data are shown as mean ± SD, *n* = 6 in each group; *p* < 0.01, d day)

Preoperative physiological and behavior baseline were defined by using the average body weight values, duration time, frequency of the face grooming and the time of desperate resting behavior from the last 3 days, and the average water and food intake measures from the last 2 days before the surgery. These parameters were continued to be recorded every day from 1 to 14 days of operation [8] (Fig. 1A).

### HE staining and immunostaining

We extracted the left maxillary first molars of rats at each time period and performed hematoxylin and eosin (HE) staining to observe the progress of inflammation, then cut the medulla oblongata and the cACC tissues for immunostaining and immunofluorescence staining, respectively.

### Western blot

The total protein of cACC tissue was extracted to conduct western blotting assay. Total protein extracts (25 µg) were loaded to perform western blotting analysis with antibody against NR2B and p-NR2B. Protein bands were detected by enhanced chemiluminescence (ECL, Millipore, USA) and imaged with a Bio-Rad chemiDoc XRS + imaging system (USA) [9].

### PET imaging and image analysis

In vivo 2-deoxy-2-[^18^F] fluoro-D-glucose (^18^F-FDG) PET scanning was performed at different time periods after DPI surgery. The images were reconstructed using a modified back projection algorithm and analyzed using the AMIDE (version 9.2; Stanford University) and Statistical Parametric Mapping (SPM) software. We adopted two independent *t*-tests to evaluate regional metabolic differences between baseline and post-stimulation PET images. Statistical significance was determined when *p* value < 0.01 and cluster *Ke* > 100 [10]. The lesion-to-normal (L/N) ratio was used for semi-quantitative analysis by using PMOD (v.3.902, PMOD Technologies Ltd.). The regional cerebral metabolism rate (rCMR) of each ROI was calculated as the lesion-to-pons (L/P) ratio.

### Statistical analysis

Data were expressed as mean ± SD. A value of *p* < 0.05 was considered as significant. Statistical analyses were performed by Student’s *t*-test and one-way and two-way analysis of variance (ANOVA) and the SPSS software (version 22.0, SPSS Inc.).

## Results

### Behavioral assessment and histology of DPI

We observed the drinking, diet, and average weight gain of DPI rats decreased greatly in comparison with the sham group, while the duration and frequency of facial grooming and the frozen time in forced swimming test (as a model of depressive-like behavior) [11] were increased significantly, especially during the first 3 days in DPI. All these parameters returned to the baseline levels on the 14th day after surgery (Fig. 1A). Notably, our results of HE staining showed that the inflammation appeared gradually from the crown to the root pulp in DPI, and the acute (days 1–3) and chronic inflammation (days 4–17) and finally dental pulp necrosis (on the 14th day) after surgery were also identified (Fig. 1B, SI Appendix, Fig. S4).

### Glucose metabolism in the rat brain after DPI

**Fig. 2:**
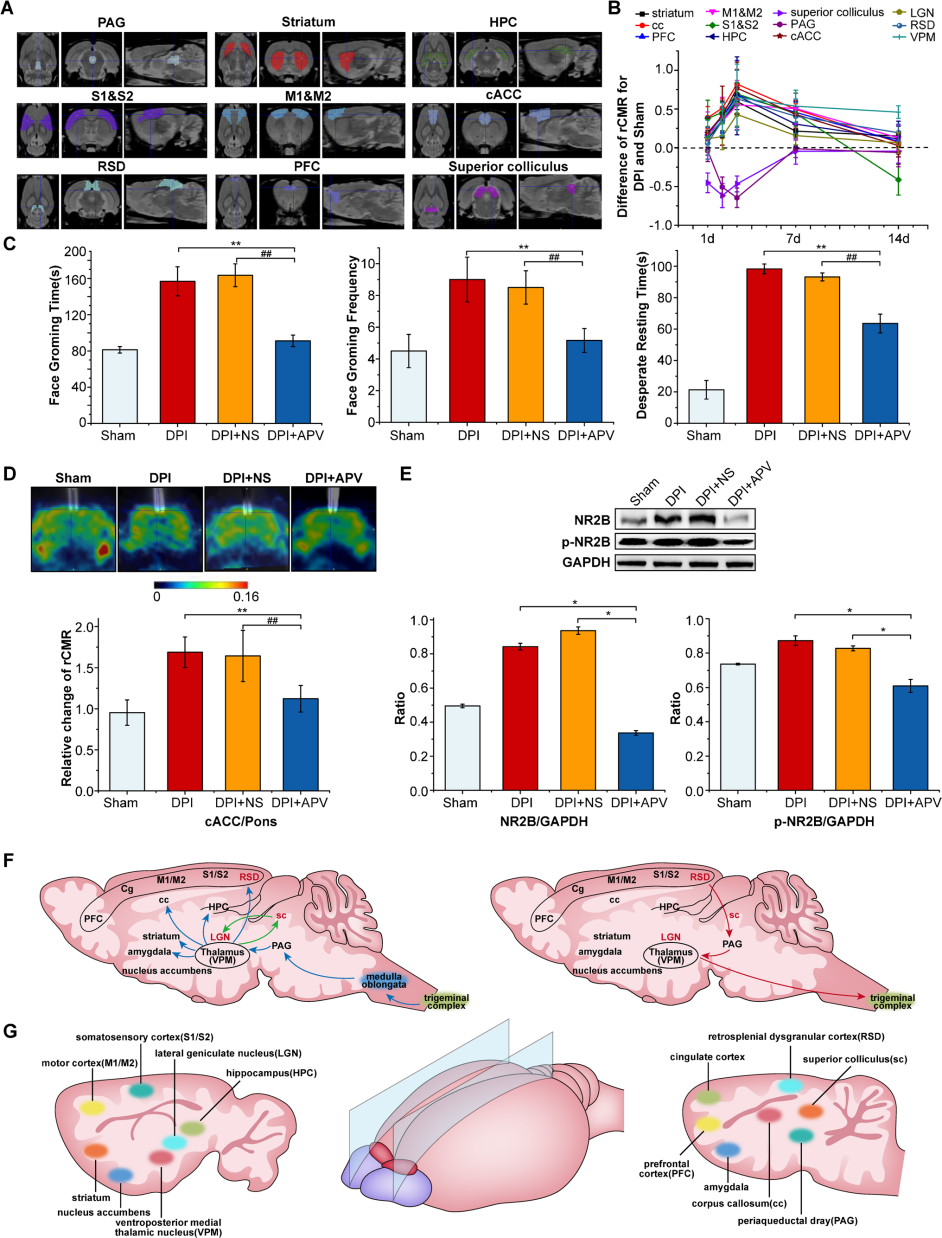
Nerve conduction pathway related to toothache in DPI. **A** The transverse (left), coronal (middle), and sagittal (right) of different brain regions of the rat using different colors, mainly including PAG, striatum, HPC, S1& S2, M1&M2, cACC, RSD, PFC, superior colliculus et al. **B** The rCMR of each ROI of the 12 brain regions after DPI by PMOD. The *p* values of relative change of rCMR in each group were shown in Table S2 (two independent samples *t*-test; *n* = 6 in each group, d day). **C**–**E** Face grooming time and frequency, and desperate resting time, glucose metabolism after injecting APV in cACC region, and the expression of NR2B and p-NR2B proteins were analyzed in DPI + APV group compared to DPI and DPI + NS groups (data are shown as mean ± SD, **, ^##^: *p* < 0.01, one-way ANOVA test. DPI dental pulp injury; NS normal saline). **F** Schematic of major ascending (bottom-up) pathways from the medulla oblongata to the brain that are activated by noxious stimuli related to toothache (left) and descending (top-down) pathways that modulate transmission of ascending nociceptive signals (right). **G** Schematic of the location of brain regions that showed significant glucose metabolism changes in sagittal view after DPI

The analysis of the SPM and PMOD results showed that the main manifestations were the increased absorption of ^18^F-FDG in the brain, including ventroposterior medial thalamic nucleus (VPM), corpus callosum (cc), hippocampus (HPC), striatum, somatosensory cortex (S1&S2), motor cortex (M1&M2), cingulate cortex (Cg), retrosplenial dysgranular cortex (RSD), prefrontal cortex (PFC), and lateral geniculate nucleus (LGN), while periaqueductal dray (PAG) and superior colliculus inhibited absorption in the process of toothache (Fig. 1C, 2A and B, SI Appendix, Fig. S2, Table S1, S2 and S3). The accumulation of ^18^F-FDG peaked on the 3rd day after DPI in a wide range of brain regions, and then decreased gradually from 7 to 14 days after DPI surgery.

### cACC participation in emotional regulation

In order to further clarify the potential contribution of cACC to toothache, APV (also known as AP5, (2R)-amino-5-phosphonovaleric acid, or (2R)-amino-5-phosphonopentanoate), a selective NMDA receptor antagonist, was applied to reduce the expression of NR2B and block the neuronal response of cACC [12]. The optimal concentration of APV was determined by autoradiography (SI Appendix, Fig. S3). After APV administration in the cACC, we observed significantly decreased duration of grooming and freezing (*p* < 0.01, Fig. 2C), reduced expression of NR2B and p-NR2B, and downregulated rate of glucose metabolism (*p* < 0.05, Fig. 2D and E).

## Discussion

The behavioral, histological, and metabolic dynamic changes of toothache were longitudinally evaluated for up to 14 days in a rat DPI model. Interestingly, a perfect correlation among the behavioral, histological examinations and PET imaging was identified during the natural process of toothache, and therefore, our study provided a comprehensive understanding for the neurofunctional patterns underlying toothache. More importantly, the nerve conduction pathways related to toothache, especially the emotional reaction to toothache are identified. The related features provide rich resource to inspect toothache-induced brain changes.

In this study, we chose the DPI model as the suitable model to investigate the natural course of toothache. The exposure in the pulp of first molars could lead to local pulp tissue infection by normal flora of the oral cavity, the increase of tissue pressure in the pulp cavity, and the release of inflammatory mediators and neuropeptides, which directly or indirectly acted on the nerve endings of the pulp tissue and resulted in the trigeminal pain symptoms. During this process, the inflammatory response was limited to the pulp cavity and root canal, and did not affect the apical tissue, trigeminal nerve, and related cerebral cortex beyond the apical foramen [13, 14]. If the DPI model is locally anesthetized, the nerve endings of the rat dental pulp tissue might be blocked and cannot receive nociceptive signals and reduce a series of pain responses. Therefore, locally anesthesia assay could be helpful to further strength our conclusions observed in DPI model.

The upload of toothache stimulation signal caused transient and large-scale reactive activation of rat brain nuclei, and there was a relationship between the functional connectivity of each nucleus and the strength of pain signals. According to previous literatures [15] and the results of the current study (Fig. 2F and G), the ascending pathway of toothache is postulated to be conducted through the trigeminal nerve complex to PAG, cc and VPM, and then to the subcortical area (nucleus accumbens, striatum, hippocampus, and amygdala) and cerebral cortex (S1&S2, M1&M2, PFC, Cg, RSD). Among them, pain signals cross cc and PAG, which activate most of the brain regions bilaterally, thus regulating the transmission of pain in the left maxillary teeth. Different from previous studies [16], the visual signals related to VPM, superior colliculus and LGN, as well as RSD may also be involved in the transmission of toothache. Among them, the superior colliculus and LGN can make a visual reflex response to tactile information [17], thus reducing perception and emotion caused by pain, suggesting that the signals of toothache might activate the eye branch when ascending through the trigeminal ganglion, which explains why some patients with acute toothache often experience symptoms of eye discomfort and even tears [18]. The RSD is involved in the control of spatial navigation, episodic memory, and pain-related emotional behaviors [19]. It is speculated that RSD might be related to the cognitive and spatial memory of toothache in rats. Here, PET showed, for the first time, that the degree of activation and inhibition of the 12 brain regions involved in toothache is closely related to the changes of its pain intensity and may also be related to the cognition and memory of toothache in rats. The changes of brain network caused by toothache are very complex and elusive and need to be further investigated.

The pons was selected as the intensity normalization region to ensure the consistency of the data. It is reported that the pons is the best brain region for the normalization of intensity on the ^18^F-FDG-PET scans of the brain [20], which can be adopted for visual and semiquantitative analysis of brain ^18^FDG-PET imaging associated with other diseases in the absence of any pathological involvement [21]. However, the current international stable and reliable region for brain PET image analysis related to endodontic pain still needs further research.

Toothache induces anxiety- and depression-related symptoms, thus may cause the patients to miss the best time window to get treatments. Therefore, it is of great significance, to understand how the toothache-related emotion was regulated in order to alleviate the negative emotions. When ectopic pain occurs, synaptic responses in the ACC are enhanced in rodent models of neuropathic pain, which is induced by long-term potentiation (LTP). And the development of LTP is relied on the NMDA receptors and their signaling pathway. APV, as a selective NMDA receptor antagonist, could prevent NMDA receptor activation-induced LTP and inhibit synaptic plasticity [9, 12]. APV administration reversed the emotional response related to toothache. In our study, the results from experimental group (DPI + APV) and control groups (DPI, DPI + NS) indicated that the changes of behavior and glucose metabolism in the cACC brain area may be due to APV acting on NMDA receptors in the cACC brain area and reducing the expression of NR2B total protein production. The decrease of total NR2B in DPI + APV group might be induced by APV administration, followed by the decrease of p-NR2B in our study. Meanwhile, we also observed that NR2B expression level was increased in DPI group compared with sham group. In a previous study, in a rat model of F-CPA (formalin-induced conditioned place avoidance), unilateral intraplantar (i.pl.) injection of dilute formalin also induced NR2B expression in rACC brain region compared with normal saline group [9]. Meanwhile, APV administration in sham rats might have no effect on the synaptic response, behavioral, and metabolic changes. Adkins et al. reported that APV administration in vehicle-treated rats had no effect on fear generalization when compared with vehicle-vehicle animals [22]. Therefore, these results suggest that cACC but not rACC may mediate the emotional reaction to toothache by NR2B receptor, which is the difference between emotional regulation of toothache and somatic pain. This might provide a novel insight for relieving the pain affect induced by toothache.

## Conclusion

Our research results indicate changes in rat brain function and toothache-related nerve conduction circuits during toothache, and we found that NR2B in cACC might mediate the aberrant emotional behaviors during the toothache natural course. This may provide a certain basis for the clinical prevention and treatment of toothache-related emotional symptoms.

## Supplementary Information

Below is the link to the electronic supplementary material.

Supplementary file1 (DOCX 11647 KB)
