# Supplementary material for: Spatiotemporal dynamics of brain function during the natural course in a dental pulp injury model
Source: Eur J Nucl Med Mol Imaging. 2022 Mar 19;49(8):2716–22. doi: 10.1007/s00259-022-05764-2 (PMC9206688; doi:10.1007/s00259-022-05764-2)
Supplement: Supplementary file 1 — Supplementary file1 (DOCX 11647 KB) [file 259_2022_5764_MOESM1_ESM.docx]

**Spatiotemporal Dynamics of Brain Function During the Natural Course in a Dental Pulp Injury Model**

Feiyan Yu ^a#^, Miao Li ^a#^, Qianqian Wang ^a#^, Jing Wang ^b#^, Shuang Wu ^b#^, Rui Zhou ^b#^, Han Jiang ^c^ , Xiaoyi Li ^b^, Yu Zhou ^a^, Xi Yang ^a^, Xiao He ^b,d,e^, Yan Cheng ^f^, Xiuyun Ren ^a*^, Hong Zhang ^b,d,e*^ and Mei Tian ^b,d,e,g*^

^*^Correspondence:

Prof. Hong Zhang, PhD, Department of Nuclear Medicine and PET Center, The Second Hospital of Zhejiang University School of Medicine, 88 Jiefang Road, Hangzhou, 310009, Zhejiang, China. Email: hzhang21@zju.edu.cn.

Prof. Xiuyun Ren, PhD, Department of Periodontology, Shanxi Medical University School and Hospital of Stomatology, No. 63, New South Road, Yingze District, Taiyuan, 030001, Shanxi, China. E-mail: rxy611@163.com.

Prof. Mei Tian, PhD, Human Phenome Institute, Fudan University, Shanghai, 201203, China.

Email: tianmei@fudan.edu.cn.

**Supplementary information – Materials and Methods, Results, Table S1, Table S2, Table S3, Figure S1, Figure S2,** **Figure S3, Figure S4** **and References**

**Materials and Methods**

**Animals and groups**

This study was performed with the approval of the Institutional Animal Care and Use Committee at Zhejiang University (protocol: ZJU20190015). All procedures and animal care were also conducted in accordance with the guidelines of the International Association for the Study of Pain. Adult male Sprague–Dawley（SD）rats (Shanghai SLAC Laboratory Animal Co., Ltd., Shanghai, China) weighting from 240 to 260 g were used in this study. The animals were housed under standard laboratory conditions with food and water ad libitum. The rats (*n* = 60) were randomly assigned to the DPI groups and Sham groups (1st, 2nd, 3rd, 7th, 14th day respectively, total of 10 groups，n = 6 in each group). For the intervention research, 48 rats were randomly divided into four groups (*n* = 12 in each group). The day of DPI was defined as the day 0.

**Experimental Dental Pulp Injury (DPI)**

We performed DPI on rat by mechanically exposing the dental pulp as previously described [1], which produces pulpal inflammation (pulpitis) followed by necrosis of the pulpal tissues. Thereinto, sham rats received anesthesia without pulp exposure surgery. All animals were returned to their home cages after recovery from anesthesia with free access to food and water. Animals with accidental damage beyond the intended pulp exposure were excluded from the study. All operations were performed by the same experienced operator with clinical training to minimize the variability derived from DPI operations.

**Stereotactic Surgery and Cannula Implantation**

According to the method developed by Paxinos and Watson (Paxinos G & Watson C, 2007), the rats were anesthetized under sterile conditions followed by using the stereotaxic coordinates to measure relative to the bregma suture and the top of level skull. A 45-gauge stainless steel double-cannula with 40-gauge stainless style plug (RWD, Shenzhen, China.) was implanted 0.5mm above the cACC injection site (AP = -1.0 mm, ML = ±0.6 mm, DV = -2.5 mm from Bregma). The cACC, midcingulate cortex, was defined as the area of cingulate gyrus superior to the body of the corpus callosum (approximately bregma 0.00 to -2.20 mm). This division is largely consistent with the regional of the rodent anterior cingulate used elsewhere [2]. Cannula was next affixed to the skull with bone screws and cranioplastic dental cement. Rats were recovered for 7-10 days before formal testing, and given to assess wound healing, recovery of body weight. All animals had recovered their normal weight and their surgical wounds were healed at the end of recovery period.

**Behavioral Assessment**

After adaptive feeding for 3 days, the food intake, water consumption and body weight and other baseline behaviors of rats were monitored for 6 days before operation and continued to be recorded every day from 1 to 14 days of operation. The rodent food pellets and pipettes containing over 100 ml of water were provided to the rats were replaced with fresh every day. We used the average body weight values from the last 3 days and the average water and food intake measures from the last 2 days before the surgery as the preoperative baseline [3] (Fig. 1A).

First, for the face grooming test, the rats were placed in a 60 × 50 cm plexiglass boxes, and after adaptation to the environment for an hour, the time and frequency of the face grooming were recorded by video tracking system for 20 min each time, which was located in a vertical position of 1 meter below the test field. Then, the forced swimming test, we prepared the swimming device, a cylindrical plexiglass container (50 cm high, 30 cm dia.), was filled 35 cm deep with tap water (24 ± 1℃). We put the rats in the container with water for 1 minute to adapt to the environment and recorded the total time of desperate resting behavior of the rat within 5 minutes. Then, the rats were removed from container and wiped dry with a towel and returned to its cage. The desperate resting behavior was defined as that the rats kept floating and trending just to remain the nose above the water. Similarly, we used the average duration the times and frequency of the face grooming and the times of desperate resting behavior from the last 3 days before the surgery as the preoperative baselines (Fig. 1A).

**Histology**

For hematoxylin and eosin (HE) staining, the left maxillary first molar of rats was removed and fixed with 4% paraformaldehyde at 4°C for 48 h. The tissues were embedded in paraffin wax, and sliced at a 4 μm. Slides underwent a standard HE staining protocol for visualization of gross histological structures.

For immunostaining, the brain tissues were quickly collected after the rat heart perfusion, and fixed with 4% paraformaldehyde at 4 °C for 24 h. The medulla oblongata was cut and embed in paraffin and cut into 4 μm sections for immunohistochemical staining. Sections were incubated with sheep anti-c-Fos peptide antibody (1:200, abcam, ab6167, Cambridge, England) at 4°C overnight, subsequently the secondary biotinylated rabbit anti-sheep antibody (abcam, ab6747, Cambridge, England) was added at a dilution of 1:400 for 2 h at room temperature. The pictures were taken by using the optical microscope (Olympus, Osaka, Japan). The number of positive cells that characterized as dark and discernible nuclear staining were count using visual analysis, and the positive rate was calculated per 1000 cells.

For immunofluorescence staining, the remaining brain tissue was immersed in gradient sucrose for dehydration, and 40 µm thick coronal sections of the brain were transversely sliced using a freezing microtome (CM1950, Leica, Germany). After blocking with 5% bovine serum albumin for 1 h at 37 ℃, sections were incubated with rabbit anti-c-Fos (1:800, synaptic system) in primary antibodies diluent (Biyuntian) overnight at 4℃, followed by staining with fluorescein isothiocyanate (FITC)-conjugated goat anti-rabbit/mouse IgG H&L (Alexa Fluor^®^488, 1:400, abcam) for 1 h at 37 °C. The sections were obtained with Leica confocal laser scanning microscope (Leica TCS SP8, Germany). Image J software was used to quantify the positive expression of cell nuclear.

**Western Blot**

Western blotting was performed as previously described [4]. The total protein of cACC tissue were extracted by adding 150 µl RIPA lysis buffer containing protease inhibitor cocktail and phosphatase inhibitor cocktail (Biyuntian, China) at a 100:1 (v/v) ratio into samples. Total protein extracts (25 µg) were loaded to perform western blotting analysis with antibody against NR2B and p-NR2B (1:1200, abcam, Cambridge, England) and GAPDH (1:1000, abcam, Cambridge, England). Protein bands were detected by enhanced chemiluminescence (ECL, Millipore, USA) and imaged with a Bio-Rad chemiDoc XRS^+^ imaging system (USA).

**^18^ F-FDG PET Imaging**

PET imaging was conducted on a small-animal micro-PET R4 device (Siemens Preclinical Solutions, Knoxville, TN, America). Rats in each group were anesthetized with isoflurane (5%) and intraperitoneally injected with ^18^F-FDG (18.5 MBq, 500 mCi) for PET. After a 40 min uptake period, the rats were sedated with isoflurane (2% in a 2.5 L/min flow of O_2_) and positioned prone on the scanner for a 10 min static acquisition.

The images were reconstructed using a modified back projection algorithm and analyzed using the AMIDE (version 9.2; Stanford University) and Statistical Parametric Mapping (SPM) software. We adopted two independent *t*-tests to evaluate regional metabolic differences between baseline and post-stimulation PET images. Statistical significance was determined when *P* value < 0.01 and cluster *Ke* > 100 [5]. The lesion-to-normal [heterogenous](javascript:;) ipsilateral (L/N) ratio was used for semi-quantitative analysis by using PMOD (v.3.902, PMOD Technologies Ltd.). The regional cerebral metabolism rate (rCMR) of each ROI was calculated as the lesion-to-pons (L/P) ratio.

**Statistical Analyses**

Data were expressed as mean with SD. A value of P < 0.05 was considered as significant. Statistical analyses were performed by Student’s t-test and one-way and two-way analysis of variance (ANOVA) and the SPSS software (version 22.0, SPSS Inc.).

**Results**

**Glucose metabolism in the rat brain after DPI**

Compared with the Sham group, the analysis of the PMOD results showed that the expression of the relative changes of regional cerebral metabolic rate (rCMR) for ^18^F-FDG in the brain nuclei of the experimental groups were obvious, especially on the DPI 2nd, 3rd, 7th day, these results were basically consistent with the results of SPM analysis. These above results demonstrated that the external nociceptive stimulation in DPI could lead to the ascending transmission of pain signals and the activation of central nuclei.

**Glucose metabolism in the rat brain after DPI**

Interestingly, we noticed that the cACC belonging to the Cg was activated significantly according to the above SPM and PMOD analysis that revealed that the ^18^F-FDG uptake of bilateral cACC remarkably increased on day 3. In addition, the number of c-Fos+ cells in the nucleus of the trigeminal tract and cACC was also significantly increased in DPI versus Sham rats (SI Appendix, Fig. S1). It has reported that in animal models of somatic pain, the expression of NR2B (NMDA receptor subunit) increases, which could facilitate emotional responses to harmful stimuli.

**Table S1 Glucose Metabolism in the Rat Brain after DPI Surgeries. (Sham control vs. DPI, N=6)**

| Region | | Day 1 | Day 2 | Day 3 | Day 7 | Day 14 |
| --- | --- | --- | --- | --- | --- | --- |
| Ventroposterior medial nucleus (VPM) | | — | — | Left Increased | Left Increased | Left Increased |
| Corpus callosum (cc) | | Increased | Increased | Increased | Increased | — |
| Periaqueductal dray (PAG) | | — | Decreased | Decreased | — | — |
| Striatum | | Right Increased | Bilateral Increased | Bilateral Increased | — | — |
| Hippocampus (HPC) | CA1 | Left Increased | — | — | — | — |
|  | CA2 | Left Decreased | Bilateral Increased | — | Left Decreased | Right Decreased |
|  | CA3 | — | — | Left Increased | Left Increased | — |
| Cerebral cortex | Primary somatosensory cortex (S1) | Increased bilaterally | Increased bilaterally | Increased bilaterally | Increased bilaterally | Right Decreased |
|  | Second somatosensory cortex (S2) | — | Increased bilaterally | Increased bilaterally | — | — |
|  | Motor cortex (M1&M2) | Increased bilaterally | Increased bilaterally | Increased bilaterally | Increased bilaterally | — |
|  | Caudal anterior cingulate cortex (cACC) | — | Increased bilaterally | Increased bilaterally | Increased bilaterally | — |
|  | Retrosplenial dysgranular cortex (RSD) | — | Increased bilaterally | Increased bilaterally | Increased bilaterally | — |
|  | Prefrontal cortex (PFC) | — | Increased bilaterally | Increased bilaterally | — | — |
| Superior colliculus | | Right Decreased | Decreased bilaterally | Left Decreased | — | — |
| Lateral geniculate nucleus (LGN) | | — | — | Right Increased | — | — |

—: No changes of glucose metabolism after DPI Operation.

**Table S2 The *P.* value of PMOD analysis in different regions after DPI (Sham control *vs.* DPI, N = 6).**

| **Brain region** | **Day 1** | | **Day 2** | | **Day 3** | | **Day 7** | | **Day 14** | |
| --- | --- | --- | --- | --- | --- | --- | --- | --- | --- | --- |
|  | Mean ± SD | *P*. | Mean ± SD | *P*. | Mean ± SD | *P*. | Mean ± SD | *P*. | Mean ± SD | *P*. |
| **Increased** |  |  |  |  |  |  |  |  |  |  |
| Primary/Second somatosensory cortex (S1&S2) | 0.38±0.24 | 0.01 | 0.45±0.14 | 0.0001 | 0.69±0.15 | 0.0001 | 0.44±0.26 | 0.001 | -0.41±0.20 | 0.0001 |
| Striatum | 0.12±0.11 | 0.05 | 0.33±0.12 | 0.0001 | 0.60±0.10 | 0.0001 | 0.21±0.20 | 0.06 | 0.15±0.13 | 0.02 |
| Hippocampus (HPC) | 0.13±0.13 | 0.06 | 0.36±0.23 | 0.0001 | 0.67±0.50 | 0.006 | 0.30±0.28 | 0.01 | 0.11±0.23 | 0.20 |
| Motor cortex (M1&M2) | 0.18±0.28 | 0.19 | 0.34±0.31 | 0.02 | 0.55±0.32 | 0.0001 | 0.51±0.20 | 0.003 | 0.13±0.16 | 0.13 |
| Prefrontal cortex (PFC) | 0.18±0.22 | 0.05 | 0.40±0.20 | 0.001 | 0.68±0.45 | 0.001 | 0.46±0.14 | 0.0001 | 0.08±0.20 | 0.39 |
| Caudal anterior cingulate cortex (cACC) | 0.19±0.20 | 0.03 | 0.41±0.38 | 0.007 | 0.77±0.33 | 0.0001 | 0.43±0.18 | 0.003 | 0.03±0.15 | 0.76 |
| Corpus callosum (cc) | 0.39±0.14 | 0.0001 | 0.51±0.1 | 0.0001 | 0.82±0.19 | 0.0001 | 0.50±0.07 | 0.0001 | 0.07±0.04 | 0.01 |
| Lateral geniculate nucleus (LGN) | 0.12±0.16 | 0.04 | 0.14±0.17 | 0.03 | 0.43±0.15 | 0.0001 | 0.16±0.12 | 0.02 | 0.06±0.14 | 0.39 |
| Retrosplenial dysgranular cortex (RSD) | 0.06±0.21 | 0.44 | 0.35±0.17 | 0.0001 | 0.63±0.37 | 0.0001 | 0.41±0.22 | 0.001 | 0.19±0.11 | 0.02 |
| Ventroposterior medial thalamic nucleus (VPM) | 0.13±0.22 | 0.17 | 0.36±0.22 | 0.005 | 0.65±0.43 | 0.002 | 0.54±0.21 | 0.0001 | 0.46±0.08 | 0.0001 |
| **Decreased** |  |  |  |  |  |  |  |  |  |  |
| Superior colliculus | -0.45±0.13 | 0.0001 | -0.62±0.16 | 0.0001 | -0.47±0.10 | 0.0001 | -0.05±0.17 | 0.47 | -0.04±0.15 | 0.42 |
| Periaqueductal dray (PAG) | -0.04±0.14 | 0.48 | -0.51±0.13 | 0.0001 | -0.65±0.13 | 0.0001 | -0.01±0.11 | 0.81 | -0.07±0.18 | 0.36 |

Two independent samples T test was used.

**Table S3 Significant Glucose Metabolism changes after DPI Operation. (DPI *vs.* Sham control, N = 6)**

| **Region** | | | **Coordinate(mm)** | | | **P(unc)** |
| --- | --- | --- | --- | --- | --- | --- |
|  |  |  | **X** | **Y** | **Z** |  |
| **Day 1** | Increased | corpus callosum | -0 | 3 | -4 | 0.002 |
|  |  | Left field CA1 of the hippocampus | -2 | 3 | -4 | 0.003 |
|  |  | Left primary somatosensory cortex | -4 | 3 | -3 | 0.005 |
|  |  | Right primary somatosensory cortex | 4 | 1 | -1 | 0.009 |
|  |  | Right striatum | 2 | 8 | -2 | 0.005 |
|  | Decreased | Left field CA2 of the hippocampus | -4 | 4 | -4 | 0.004 |
|  |  | Right deep white layer of the superior colliculus | 1 | 5 | -6 | 0.006 |
| **Day 2** | Increased | Left field CA2 of the hippocampus | -4 | 5 | -6 | 0.003 |
|  |  | Right field CA2 of the hippocampus | 4 | 7 | -5 | 0.008 |
|  |  | Left primary somatosensory cortex, barrel field | -5 | 3 | -1 | 0.001 |
|  |  | Left primary somatosensory cortex, forelimb region | -3 | 0 | 1 | 0.001 |
|  |  | Right primary somatosensory cortex, upper lip region | 5 | 3 | 0 | 0.001 |
|  |  | Left primary motor cortex | -2 | 2 | 1 | 0.001 |
|  |  | Right primary motor cortex | 2 | 3 | 1 | 0.001 |
|  |  | Left striatum | -5 | 5 | -2 | 0.001 |
|  |  | Right striatum | 4 | 4 | -2 | 0.001 |
|  |  | prefrontal cortex | 0 | 3 | 1 | 0.005 |
|  |  | Right primary motor cortex | 2 | 0 | 0 | 0.001 |
|  |  | cingulate cortex | 0 | 2 | 0 | 0.001 |
|  |  | retrosplenial dysgranular cortex | 0 | 2 | -2 | 0.001 |
|  | Decreased | periaqueductal dray | 0 | 5 | -7 | 0.009 |
|  |  | deep gray layer of the superior colliculus | 0 | 4 | -7 | 0.005 |
|  |  | Left intermediate gray layer of the superior colliculus | -1 | 4 | -6 | 0.001 |
| **Day 3** | Increased | Left field CA3 of the hippocampus | -5 | 6 | -5 | 0.001 |
|  |  | Left primary somatosensory cortex | -5 | 2 | 0 | 0.001 |
|  |  | Right primary somatosensory cortex | 5 | 4 | -1 | 0.001 |
|  |  | Left primary motor cortex | -2 | 2 | -3 | 0.008 |
|  |  | Right primary motor cortex | 2 | 2 | -3 | 0.007 |
|  |  | Left striatum | -4 | 5 | -1 | 0.001 |
|  |  | Right striatum | 5 | 6 | -2 | 0.001 |
|  |  | prefrontal cortex | 0 | 4 | 0 | 0.009 |
|  |  | cingulate cortex | 0 | 2 | -1 | 0.001 |
|  |  | retrosplenial dysgranular cortex | 0 | 2 | -3 | 0.005 |
|  |  | Left ventroposterior medial nucleus | -2 | 6 | -3 | 0.002 |
|  |  | Right lateral geniculate nucleus | 4 | 5 | -5 | 0.009 |
|  | Decreased | periaqueductal dray | 0 | 5 | -6 | 0.003 |
|  |  | intermediate gray layer of the superior colliculus | 0 | 4 | -6 | 0.008 |
|  |  | Left deep gray layer of the superior colliculus | -2 | 6 | -7 | 0.007 |
|  |  | Left choroid plexus | -4 | 4 | -3 | 0.003 |
| **Day 7** | Increased | Left field CA3 of the hippocampus | -4 | 7 | -5 | 0.006 |
|  |  | Right primary somatosensory cortex | 3 | 0 | -1 | 0.001 |
|  |  | Left primary motor cortex | -1 | 0 | -3 | 0.001 |
|  |  | Right primary motor cortex | 2 | 0 | -3 | 0.001 |
|  |  | cingulate cortex | -1 | 2 | -2 | 0.009 |
|  |  | retrosplenial dysgranular cortex | 0 | 2 | -3 | 0.009 |
|  |  | Left ventroposterior medial nucleus | -3 | 6 | -4 | 0.001 |
|  | Decreased | Left primary somatosensory cortex, barrel field | -5 | 2 | -4 | 0.009 |
| **Day 14** | Increased | Left ventroposterior medial nucleus | -2 | 7 | -2 | 0.002 |
|  | Decreased | Right primary somatosensory cortex, barrel field | 6 | 4 | -1 | 0.008 |
|  |  | Right field CA2 of the hippocampus | 4 | 4 | -4 | 0.005 |

**
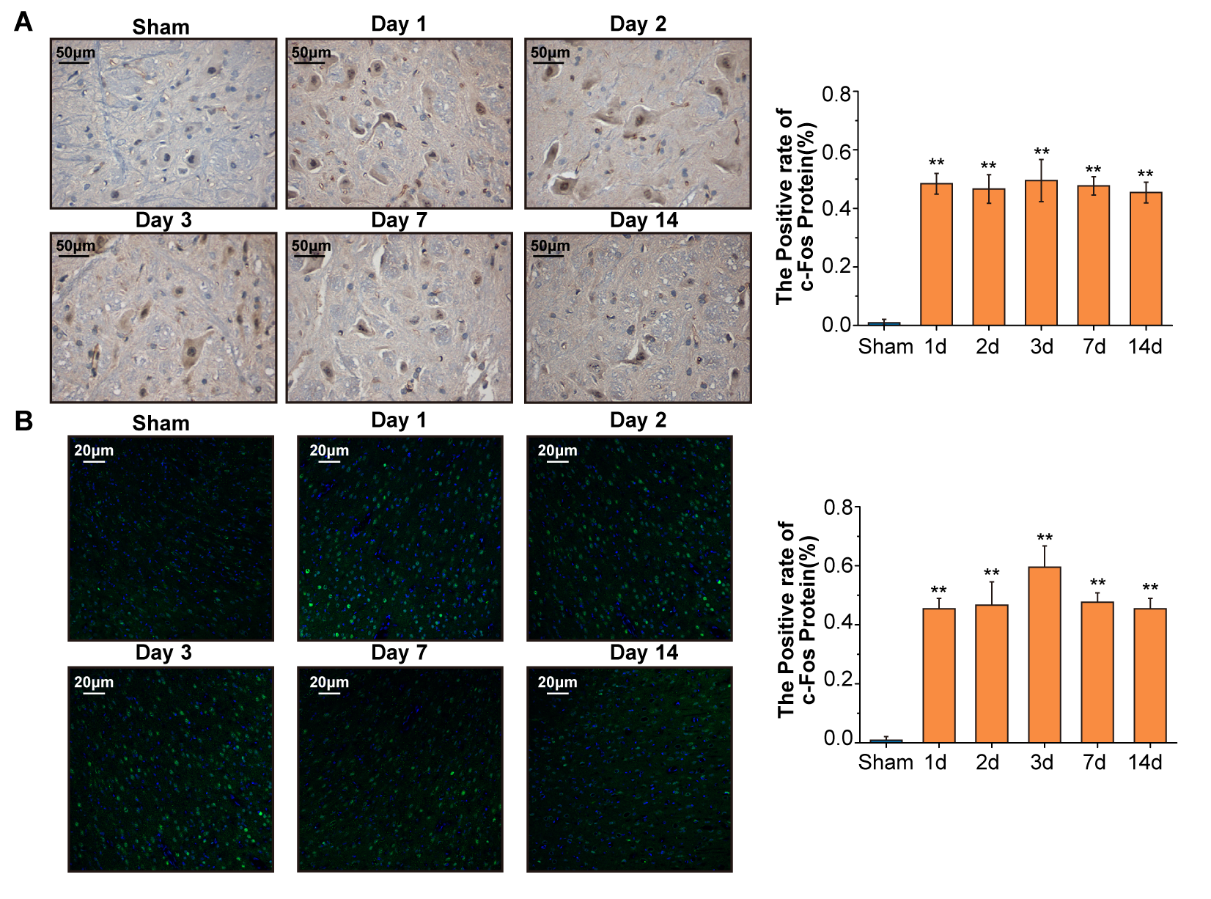
**

**Supplementary Fig. 1.** Expression of c-Fos in the rat brains after DPI. (A) The results of c-Fos protein in medulla oblongata after DPI by HE staining. Compared to the Sham group, the immunoreactivity positive cells of c-Fos protein significantly increased up to 3 days after DPI with quantification analysis (Bar = 50 μm). (B) Expression of c-Fos protein in cACC after DPI by immunofluorescence staining (Bar = 20μm). The histogram showed the quantification of c-Fos protein in cACC after DPI. (**: *P* < 0.01, *: *P* < 0.05, DPI *vs*. Sham, one-way ANOVA. *n* = 6 in each group).

**
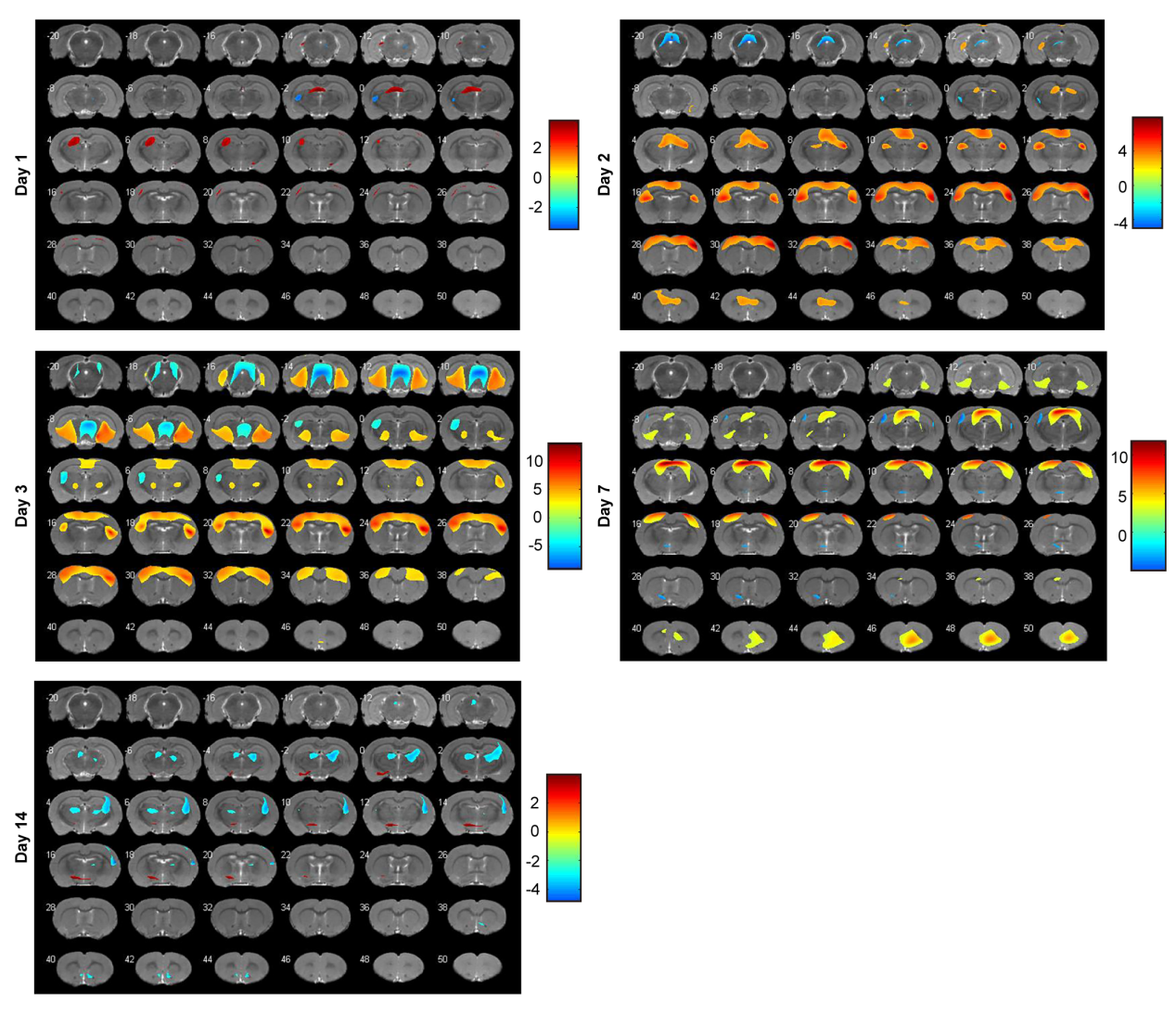
**

**Supplementary Fig. 2.** Representative PET images showing increase or decrease of glucose metabolism of the rat brain after DPI. Serial coronal images demonstrated that the range of brain regions that exhibited glucose metabolism gradually increased and then decreased. It reached the peak on the day 3 in DPI, which showed the widest range. And then the number and range of activated nuclei gradually decreased on day 7 and 14 in DPI, especially only a small part of the nucleus showed an increase or decrease ^18^F-FDG uptake on day 14 in DPI (*P* < 0.01, n = 6).


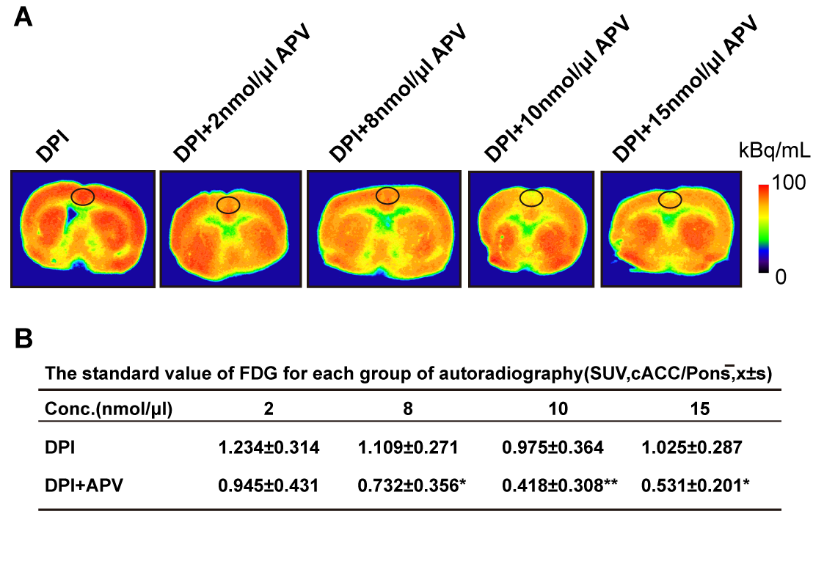


**Supplementary Fig. 3.** Autoradiographic imaging of the rat brains after APV administration. (A) Glucose metabolic changes of the cACC region after injection of different APV concentration were indicated in black circles. (B) The standard uptake value of ^18^F-FDG for each group (*: *P* < 0.05, **: *P* < 0.01).


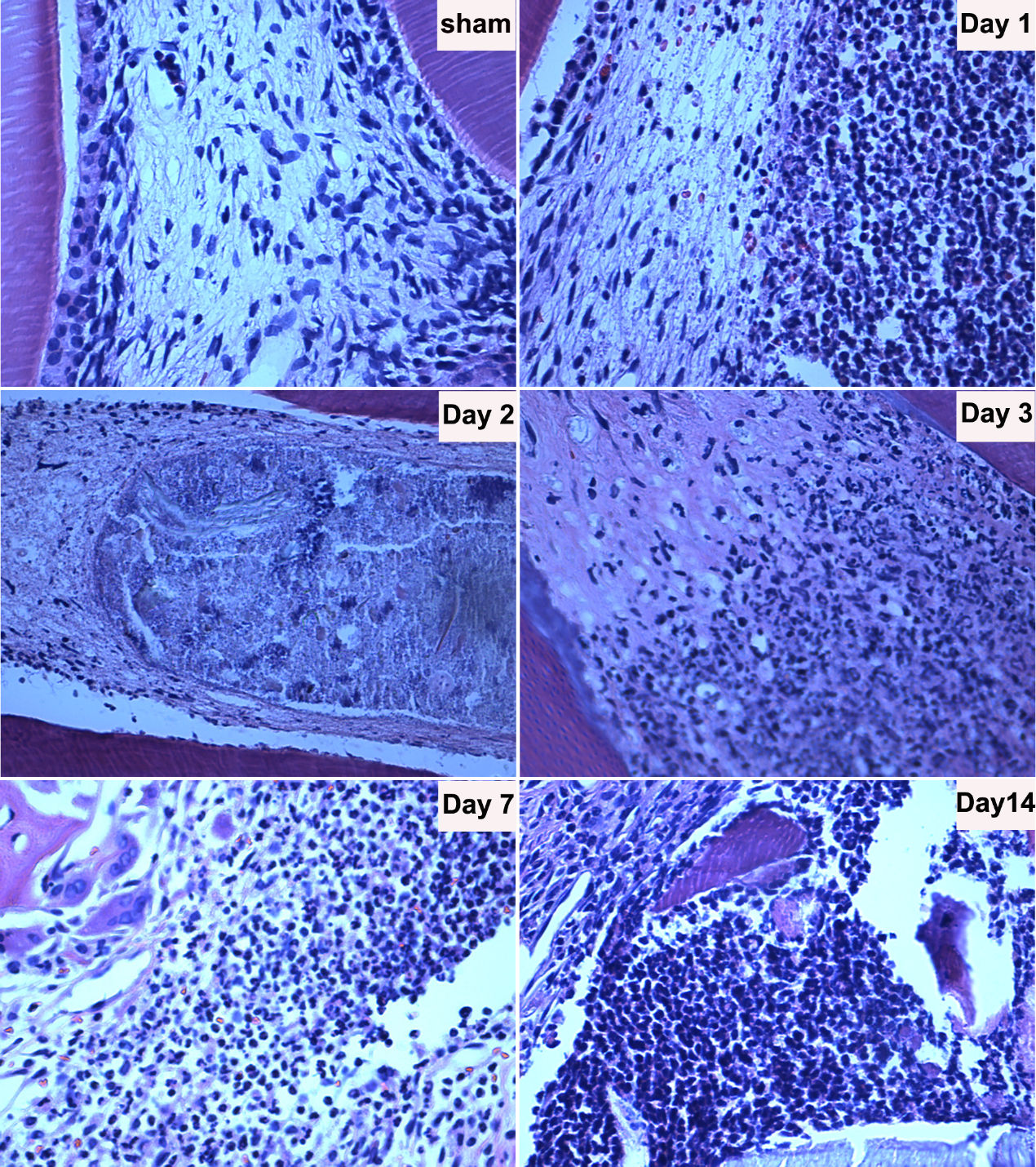


**Supplementary Fig. 4.** The high-resolution HE staining images (400×) showed the development changes of pulpitis during the nature course of DPI, including the coronal pulpal tissues 1 day after DPI, the degradation of the radicular pulp did not occur until 2-14 days after pulp exposure.

**References**

1. Haas ET, Rowland K, Gautam M. Tooth injury increases expression of the cold sensitive TRP channel TRPA1 in trigeminal neurons. Arch Oral Biol. 2011;56(12):1604-9.

2. Rozeske RR, Jercog D, Karalis N, Chaudun F, Khoder S, Girard D, et al,. Prefrontal-Periaqueductal Gray-Projecting Neurons Mediate Context Fear Discrimination. Neuron. 2018;97(4):898-910.

3. Shang L, Xu TL, Li F, Su J, Li WG. Temporal dynamics of anxiety phenotypes in a dental pulp injury model. Mol Pain. 2015;11:40.

4. Li TT, Ren WH, Xiao X, Nan J, Cheng LZ, Zhang XH, et al,. NMDA NR2A and NR2B receptors in the rostral anterior cingulate cortex contribute to pain-related aversion in male rats. Pain. 2009;146(1-2):183-93.

5. He X, Jin C, Ma M, Zhou R, Wu S, Huang H, et al,. PET imaging on neurofunctional changes after optogenetic stimulation in a rat model of panic disorder. Front Med. 2019;13(5):602-609.
